# A novel method for Pu-erh tea face traceability identification based on improved MobileNetV3 and triplet loss

**DOI:** 10.1038/s41598-023-34190-z

**Published:** 2023-04-28

**Authors:** Zhe Zhang, Xinting Yang, Na Luo, Feng Chen, Helong Yu, Chuanheng Sun

**Affiliations:** 1 National Engineering Research Center for Information Technology in Agriculture, Beijing, 100097 China; 2 College of Information Technology, Jilin Agricultural University, Changchun, 130118 China; 3 National Engineering Laboratory for Agri-Product Quality Traceability, Beijing, 100097 China

**Keywords:** Computer science, Information technology

## Abstract

Ensuring the traceability of Pu-erh tea products is crucial in the production and sale of tea, as it is a key means to ensure their quality and safety. The common approach used in traceability systems is the utilization of bound Quick Response (QR) codes or Near Field Communication (NFC) chips to track every link in the supply chain. However, counterfeiting risks still persist, as QR codes or NFC chips can be copied and inexpensive products can be fitted into the original packaging. To address this issue, this paper proposes a tea face verification model called TeaFaceNet for traceability verification. The aim of this model is to improve the traceability of Pu-erh tea products by quickly identifying counterfeit products and enhancing the credibility of Pu-erh tea. The proposed method utilizes an improved MobileNetV3 combined with Triplet Loss to verify the similarity between two input tea face images with different texture features. The recognition accuracy of the raw tea face dataset, ripe tea face dataset and mixed tea face dataset of the TeaFaceNet network were 97.58%, 98.08% and 98.20%, respectively. Accurate verification of tea face was achieved using the optimal threshold. In conclusion, the proposed TeaFaceNet model presents a promising approach to enhance the traceability of Pu-erh tea products and combat counterfeit products. The robustness and generalization ability of the model, as evidenced by the experimental results, highlight its potential for improving the accuracy of Pu-erh tea face recognition and enhancing the credibility of Pu-erh tea in the market. Further research in this area is warranted to advance the traceability of Pu-erh tea products and ensure their quality and safety.

## Introduction

Pu-erh tea is a highly distinctive tea product in Yunnan Province, China. The quality of Pu-erh tea is affected by packaging, production, processing, and storage. Different regions, varieties, and processing techniques result in different values for Pu-erh tea^1^. Pu-erh tea can be classified into Pu-erh raw tea and Pu-erh ripe tea based on processing technology^2^. Furthermore, the finished Pu-erh tea can be left as loose leaves or compressed into cakes or bricks to facilitate transportation and storage^3^. Typically, the longer the Pu-erh tea is stored, the higher the value. Many unscrupulous enterprises and individuals sell seconds at best quality prices, which seriously affects the Pu-erh tea sales market, can mislead consumers and negatively affect the economic benefits to consumers^4^.

To improve traceability and combat counterfeiting, various technological solutions have been proposed. For instance, a traceability system that uses bound Quick Response (QR) codes or Near Field Communication (NFC) chips could trace every link of the supply chain^5^. But, digital ID-based solutions cannot completely solve the problem of counterfeiting, as counterfeiters can still copy QR codes or NFC chips and fit cheaper products into the original packaging. One important way to enhance product traceability is to extract and use information about the unique and natural characteristics of the product^6^. In the case of Pu-erh tea, the different and unique natural textures formed when tea is compressed into cakes can be used as the basis for tea face images.

Computer vision technology has made it possible to use deep learning and image processing methods for biometric identification, including face recognition^7,8^. Many face recognition models and methods have been developed, such as DeepFace^9^, SphereFace^10^, central loss^11^, state-of-the-art face recognition models^12^, and LocalFace^13^. Similar methods have also been used in animal feature recognition tasks, such as automatic identification of individual cows^14^ and goats^15^, pig face recognition^16^, cow face recognition^17,18^, and individual egg identification^19^. We therefore speculated that biometric approaches could also be applied to the Pu-erh tea face recognition task.

The tea face recognition task can be divided into two types: tea face verification and tea face recognition. To improve the traceability of Pu-erh tea products, we proposed a Pu-erh tea face verification model, TeaFaceNet, based on an improved MobileNetV3. The model uses an attention mechanism module ECA block in the lightweight network MobileNetV3 for feature extraction to express texture features while reducing the number of parameters. Triplet Loss and Softmax are used as the loss function. Our experimental results showed that the validation accuracy of the model was higher than that of some classical convolutional neural networks (CNNs) models. Constructing a verification model can improve the traceability of Pu-erh tea and help avoid adulteration.

## Materials and methods

### Data acquisition

**Figure 1:**
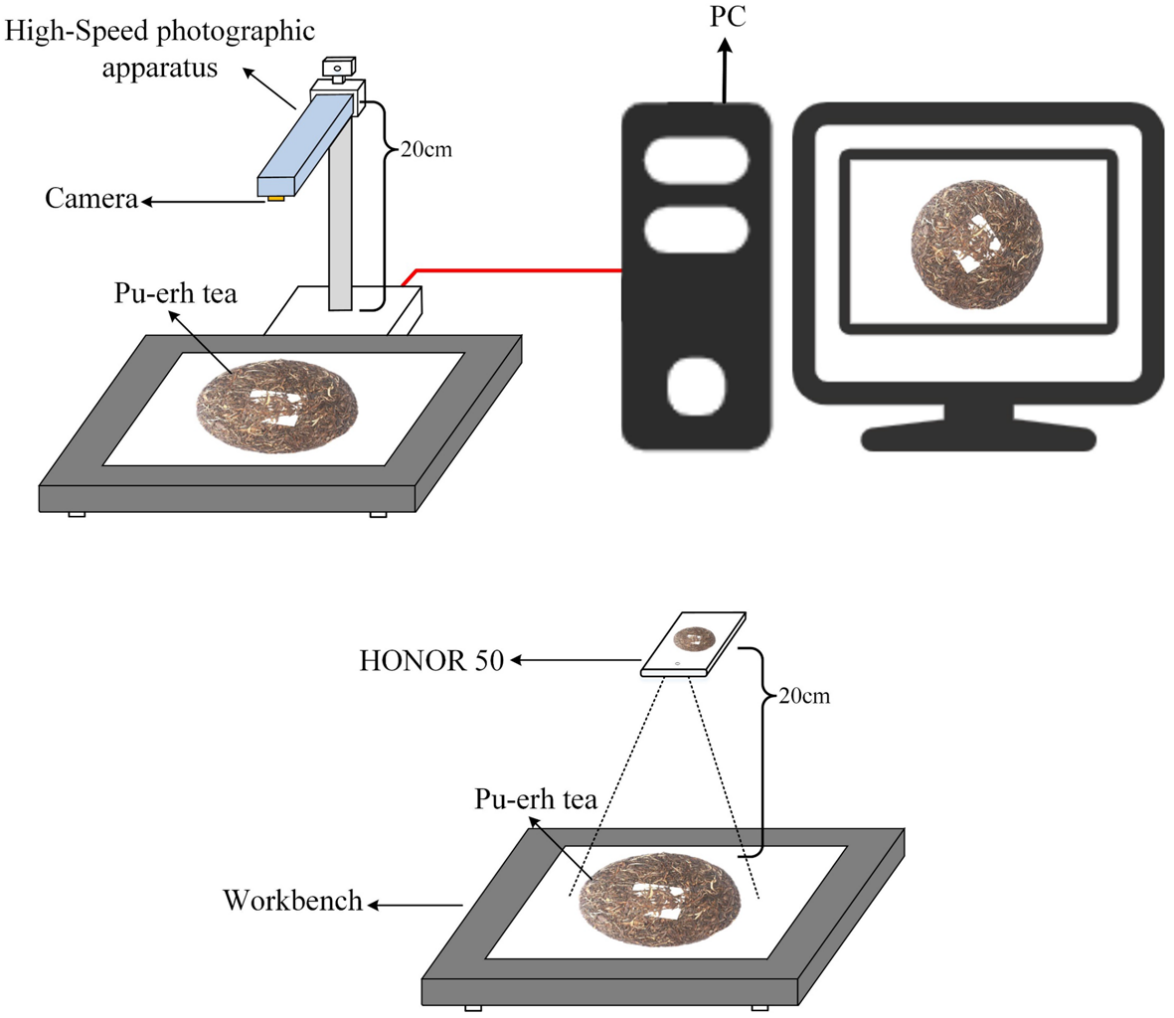
A schematic diagram of image acquisition.

The image data for this study were collected from a Pu-erh tea cake production plant in Puer city, Yunnan Province, China (22.78°N, 100.91°E). Two types of equipment were used to photograph each tea cake: a mobile phone (HONOR 50) and a High-Speed photographic apparatus (Eloam High-Speed Portable HD DocScanner S820A3AF). The purpose was to simulate real-world scenarios, and a schematic diagram of the image acquisition process is shown in Fig. 1. The Eloam High-Speed Portable HD DocScanner S820A3AF has CMOS Autofocusing technology with a 10 million pixel main camera that captures images at a resolution of 3264 × 2448. The HONOR 50 is a mobile phone released by HONOR on June 16, 2021, equipped with 108 + 8 + 2 + 2 million pixels quad cameras. The resolution of the images acquired by the mobile phone is 3904 × 2928. A total of 200 pieces of Pu-erh raw tea and 200 pieces of Pu-erh ripe tea were collected, with 100 pieces used for the training dataset and the other 100 pieces for the test dataset. Each tea cake was photographed from the front and back. The image shooting standards are as follows: (1) set off with a white background, keep the background clean and tidy without debris; (2) shoot at a distance of 20 cm directly above the tea cake; (3) ensure that the tea cake is in the center of the image; (4) make the tea cake maximally filled with pictures to ensure a clear texture.

### Preprocessing

**Figure 2:**
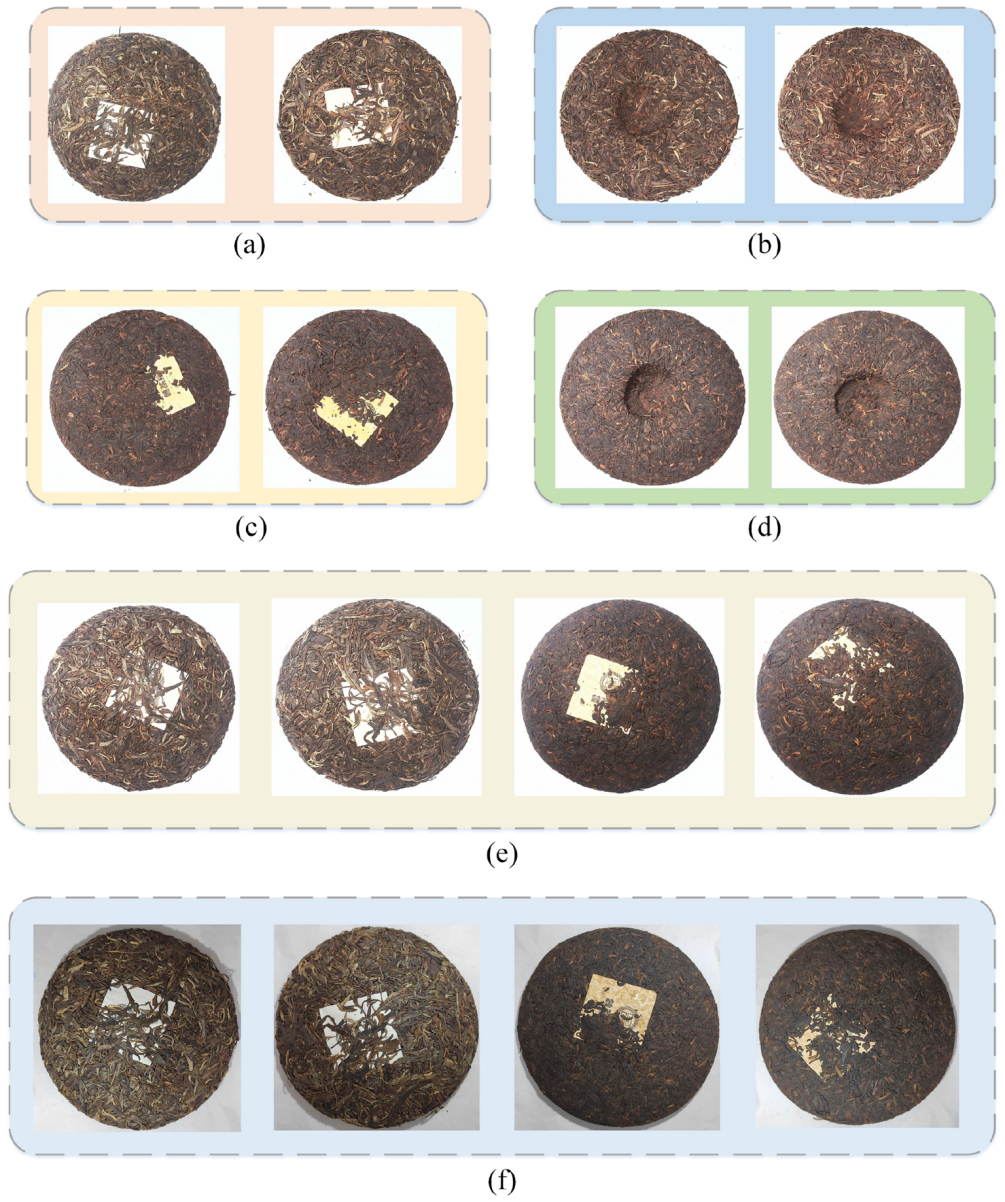
(**a**)–(**d**) are examples of Pu-erh tea face datasets. (**a**) Pu-erh raw tea face image (front), (**b**) Pu-erh raw tea face image (back), (**c**) Pu-erh ripe tea face image (front), (**d**) Pu-erh ripe tea face image (back). (**e**), (**f**) are examples of data acquired by different recording devices. (**e**) High-Speed sortable scanner, (**f**) Mobile phone.

After the data acquisition was completed, the tea cake image was processed uniformly and the resolution of the tea cake map was adjusted to 320 × 320 × 3. The images were then expanded using data enhancement techniques. After the above operations, the following three training datasets were established: Pu-erh raw tea face dataset; Pu-erh ripe tea face dataset; and mixed tea face dataset. All three datasets include the front and back images of Pu-erh raw tea and Pu-erh ripe tea. Some of the Pu-erh tea face datasets are shown in Fig. 2.

The amount of data for each training data set is shown in Table 1. The training dataset of Pu-erh raw tea faces contains 100 front and back images of Pu-erh raw tea cakes captured using two types of equipment, resulting in a total of 400 images. After applying data augmentation techniques, the total number of images increased to 8000. Similarly, the training dataset of Pu-erh ripe tea faces contains 100 front and back images of Pu-erh ripe tea cakes taken using two different devices, resulting in a total of 400 images. After data augmentation, the total number of images increased to 8000. The mixed tea face dataset contains all the raw and ripe Pu-erh tea faces from the previous datasets, resulting in a total of 800 images. After data augmentation, the total number of images increased to 16,000. During the training process, the dataset was split into training set and validation set in a 9:1 ratio. The training set and validation set for Pu-erh raw tea face dataset and Pu-erh ripe tea face dataset contained 7200 and 800 images respectively, while for Mixed tea face dataset, they contained 14,400 and 1600 images respectively.

**Table 1:** Training dataset data.

Dataset	Number of tea face	Number of tea face images	Number of images after enhancement
Pu-erh raw tea face dataset	100	400	8000
Pu-erh ripe tea face dataset	100	400	8000
Mixed tea face dataset	200	800	16,000

The test dataset was shot with the same shooting method of 100 pieces each of Pu-erh raw tea and Pu-erh ripe tea, containing both front and back images, as shown in Table 2. Among them, 1200 test pairs (600 pairs of the same tea face and 600 pairs of different tea face) were selected for each of the Pu-erh raw tea face test dataset and the Pu-erh ripe tea face test dataset, and 2400 test pairs (1200 pairs of the same tea face and 1200 pairs of different tea face) were selected for the mixed tea face dataset.

**Table 2:** Test dataset data.

Dataset	Number of tea face	Number of tea face images	Number of test pairs
Pu-erh raw tea face dataset	100	400	1200
Pu-erh ripe tea face dataset	100	400	1200
Mixed tea face dataset	200	800	2400

### Data enhancement

**Figure 3:**
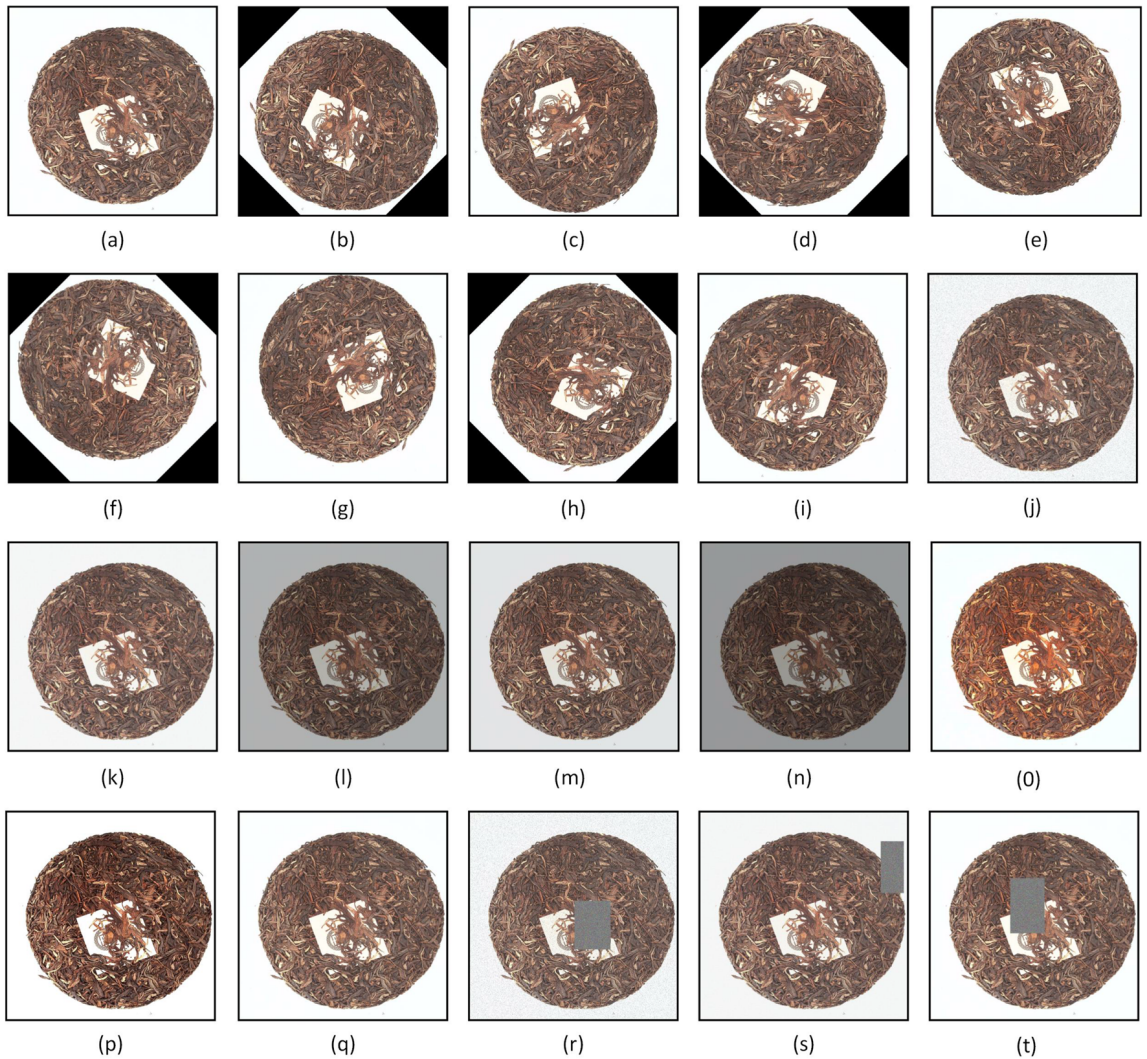
Data enhancement methods, (**a**) original image, (**b**) 45° clockwise rotation, (**c**) 90° clockwise rotation, (**d**) 135° clockwise rotation, (**e**) 180° clockwise rotation, (**f**) 225° clockwise rotation, (**g**) 270° clockwise rotation, (**h**) 315° clockwise rotation, (**i**) mirror flip, (**j**) salt-and-pepper noise, (**k**) Gaussian noise, (**l**), (**m**), (**n**) random brightness adjustment, (**o**), (**p**), (**q**) random adjustment of chroma, contrast and sharpness, (**r**), (**s**), (**t**) random erasing.

When photographing the tea cake, it is difficult to determine a fixed direction due to its round shape. To improve the robustness of the deep neural network for tea face recognition in various scenes, we used rotation, flipping, random contrast and brightness adjustments, image noise, and random erasing to enhance the data. This data augmentation technique enriches the dataset and improves the generalization of the model, allowing it to learn enough features to enhance its performance. The data enhancement techniques are illustrated in Fig. 3.

### Image rotation

Firstly, image enhancement was performed using rotation. Rotate the original image by 45°, 90°,135°, 180°, 225°, 270°, and 315° while performing one mirror flip. This was done so that the model could learn the features of each angle and improve the rotation invariance of the model.

### Image noise

In terms of image noise, Salt-and-pepper noise and Gaussian noise were used to enhance the image data. Salt-and-pepper noise is a very important noise, which mainly changes pixels to black and white randomly^20^. Compared with other noises, images are more sensitive to salt-and-pepper noise. Gaussian noise, which is a noise whose distribution obeys a normal distribution, is superimposed on every point of the image. Using these two methods to enhance the image could improve the ability of the model to mine the deep features of the image and enhance the recognition performance of the model in complex scenes.

### Image brightness, chroma, contrast, sharpness

In terms of image brightness adjustment, the following measures were used to enhance the data. Adjusts the brightness of the original image by selecting three random values, and these three random values were constrained to a range, namely ${\mathrm{Value}}_{\mathrm{min}}=0.5$ ${\mathrm{Value}}_{\mathrm{min}}=0.5$ and ${\mathrm{Value}}_{\mathrm{max}}=2.0$ ${\mathrm{Value}}_{\mathrm{max}}=2.0$ ${\mathrm{Value}}_{\mathrm{max}}=2.0$ ${\mathrm{Value}}_{\mathrm{max}}=2.0$. In the image chromaticity, contrast, and sharpness adjustment, the same measures were taken to enhance the data. After the enhancement adding the images to the training set, the main purpose of this enhancement method is that it can simulate the situation under different light intensities when the tea face was taken. Also, the data processed by this method could make up for the shortcomings of the neural network and make it more robust when testing the data under different light intensities.

### Image random erasing

Zhun Zhong et al.^21^ proposed a random erasure method for training CNNs that randomly selects rectangular regions in an image while modifying their pixels using random values. By using this method, images with different occlusion levels could be generated, which could reduce the risk of overfitting and make the model robust to occlusion.

### Lightweight network MobileNetV3

MobileNet^22^ was a lightweight network designed for mobile devices and embedded devices. Nowadays, the available versions include MobileNet, MobileNetV2^23^, and MobileNetV3^24^. MobileNetV3 combines the structures in MobileNet and MobileNetV2, while it introduces the Squeeze-and-Excitation (SE) block^25^.

**Figure 4:**
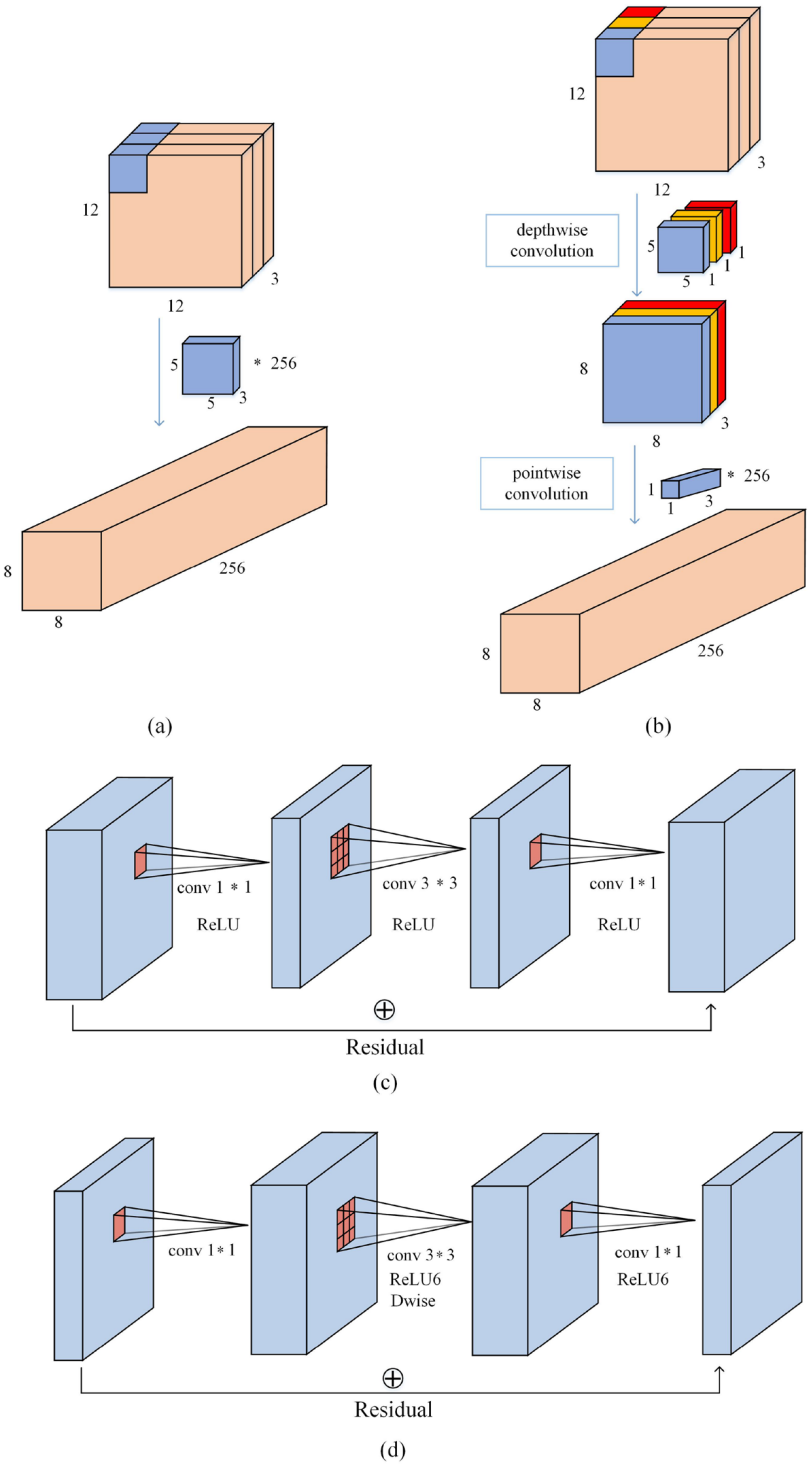
(**a**) Traditional convolution; (**b**) Depthwise separable convolution; (**c**) Residual block; (**d**) Inverted residuals and linear bottlenecks.

Firstly, MobileNetV3 used depthwise separable convolution, which was designed to reduce the amount of computation and improve the computational speed of the network. Depthwise separable convolution mainly includes depthwise convolution and pointwise convolution. The depthwise convolution was to change the convolution kernel in the standard convolution into a single-channel convolution kernel. When the input had N number of channels, there will be N single-layer convolution kernels, and each channel was convolved separately and finally superimposed. Pointwise convolution was used to expand the channels by using 1 × 1 convolution. A comparison with standard convolution is shown in Fig. 4a and b.

Secondly, MobileNetV3 used linear bottleneck, Expansion layer and Inverted residuals. The linear bottleneck was used to reduce the loss of feature information, and the inverted residuals were used to learn more features by expanding the channels. The residual block was by descending and then ascending, while the inverted residual block was by ascending and then descending. Figure 4c shows the residual blocks, and Fig. 4d shows the inverted residuals and linear bottlenecks.

**Figure 5:**
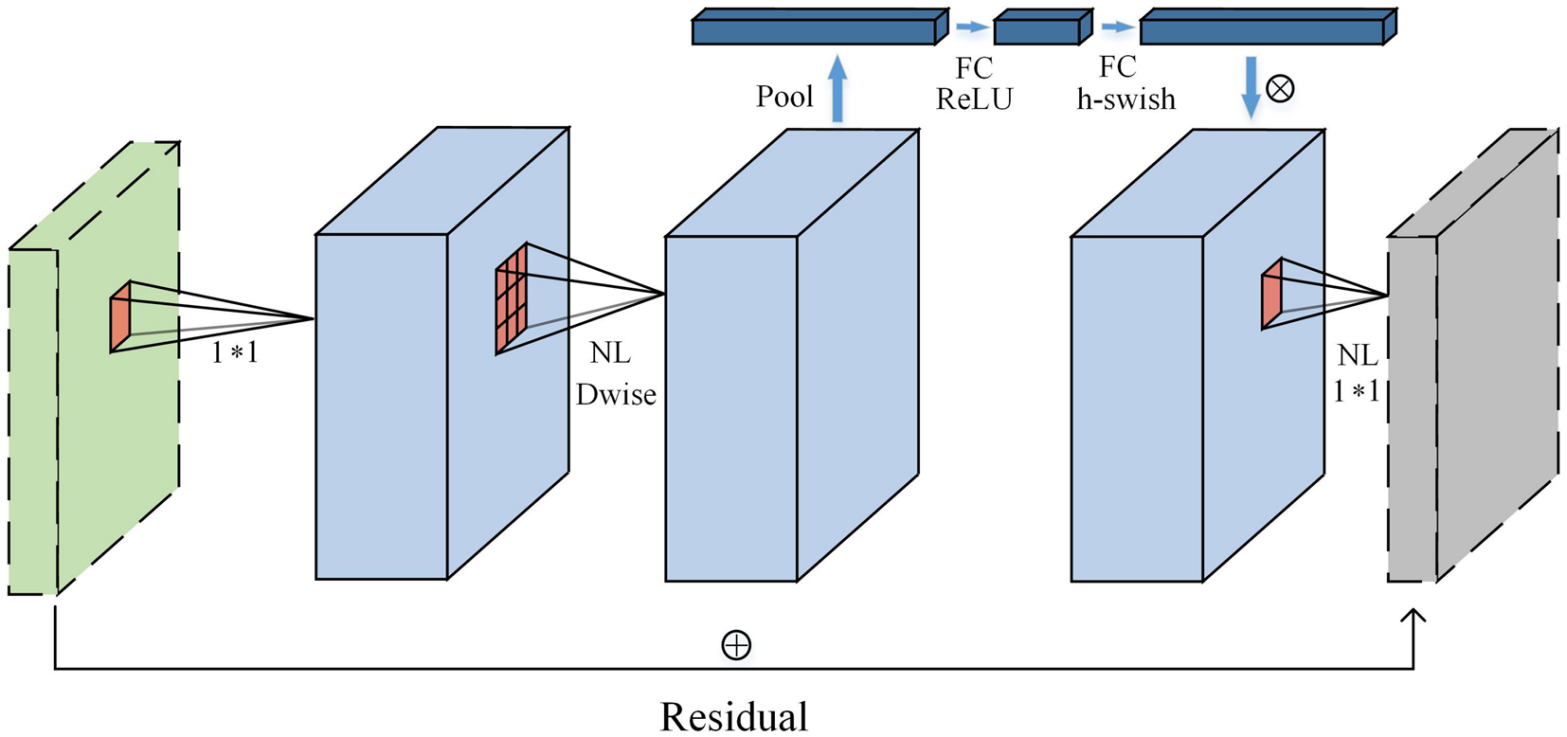
MobileNetV3 block, the symbols and ⊕ indicate the connection operation and the sum of elements.

Finally, MobileNetV3 placed the lightweight attention model of the squeeze and excitation structure after the depth filter in the extension in order to facilitate the application of attention to the largest representation. Figure 5 shows the structure of the MobileNetV3 block and a new activation function $h{ - }swish\left[ x \right]$ is used. The $h{ - }swish\left[ x \right]$ is shown in Eq. (1).

1 $$ h{ - }swish\left[ x \right]{ = }x\frac{{{\text{ReLU6(}}x{ + 3)}}}{{6}} $$

### Attention mechanism module

Attention mechanisms were essentially a set of weighting coefficients learned autonomously by the network and "dynamically weighted" to emphasize regions of interest while suppressing irrelevant background regions. The mainstream attention mechanisms include channel attention and spatial attention.

**Figure 6:**
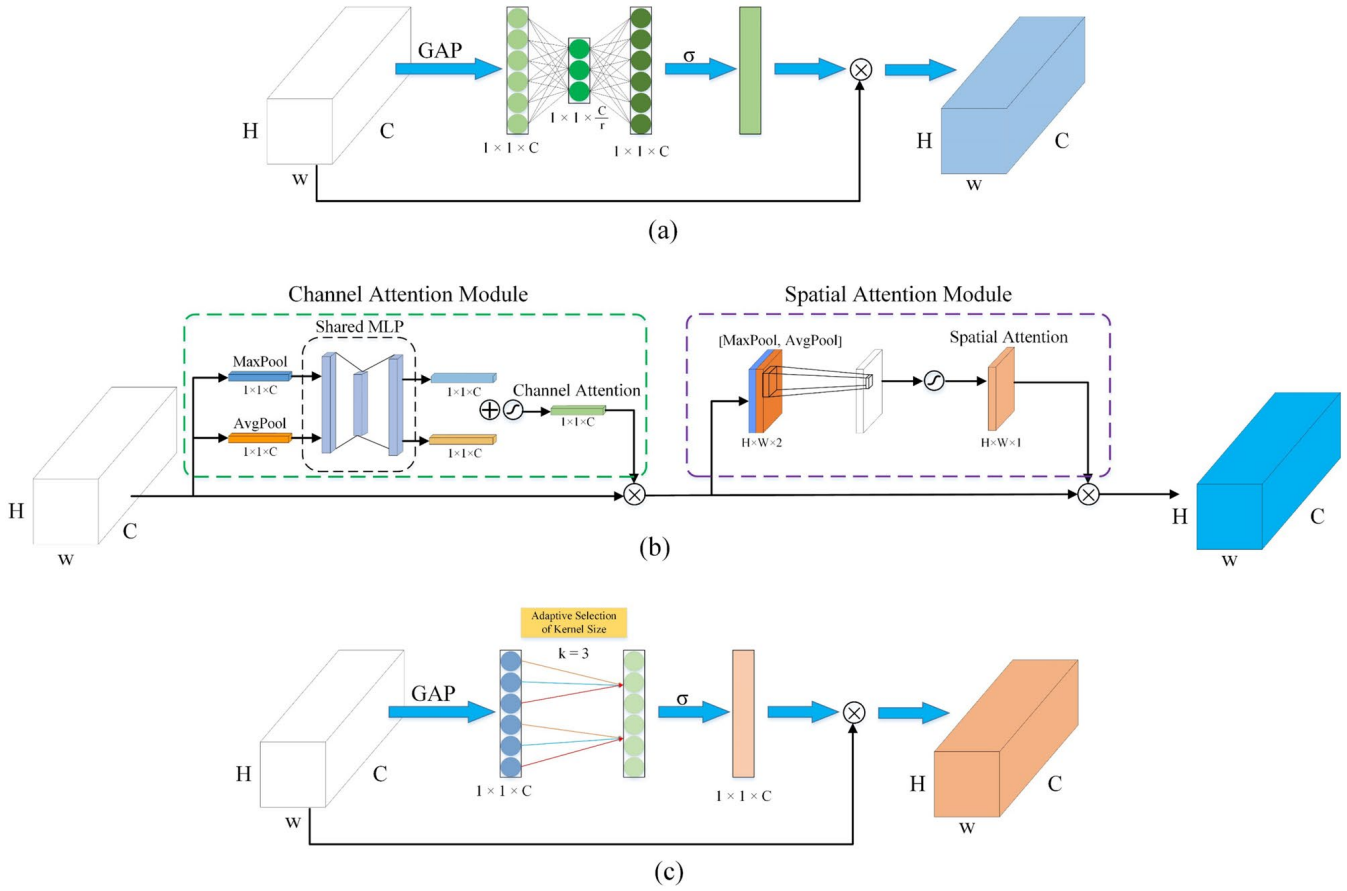
(**a**) SE block, (**b**) CBAM block, (**c**) ECA block. $W$, $H$, and $C$ are width, height, and channel dimension (i.e., number of filters).

Firstly, the squeeze and excitation(SE) block, which was the main representative of channel attention. This attention mechanism module was used in MobileNetV3. The SE block is shown in Fig. 6a. It is mainly composed of two parts: squeeze and excitation. Secondly, the convolutional block attention module (CBAM)^26^ was used in this experiment, which was based on the original channel attention and bridged with a spatial attention module (SAM). Figure 6b shows the structure of the CBAM module.

The structure of the Efficient Channel Attention (ECA) block^27^ is shown in Fig. 6c. It used a 1-dimensional sparse convolution operation to optimize the fully connected layer operations involved in the SE block to significantly reduce the number of parameters and maintain a comparable performance. In order to compress the number of parameters and improve the computational efficiency, the SE block adopts a "dimensionality reduction-then dimensionality increase" strategy, using two multilayer perceptrons to learn the correlation between different channels, i.e., each current feature map interacts with other feature maps, which is an intensive connection. ECA module simplifies this connection by making the current channel interact with its k domain channels only, aggregated features are obtained by global average pooling (GAP), and ECA generates channel weights by performing a fast $1D$ convolution of size $k$, where $k$ is determined adaptively by mapping the channel dimension $C$. The $k$ is shown in Eq. (2).

2 $$ k = \psi \left( C \right) = \left| {\frac{{log_{2} \left( C \right)}}{\gamma } + \frac{b}{\gamma }} \right| $$

where $\left| t \right|_{odd}$ represents the odd number nearest to $t$. $\gamma$ and $b$ are set to 2 and 1.

### Proposed model architecture

#### TF-Bottleneck block

**Figure 7:**
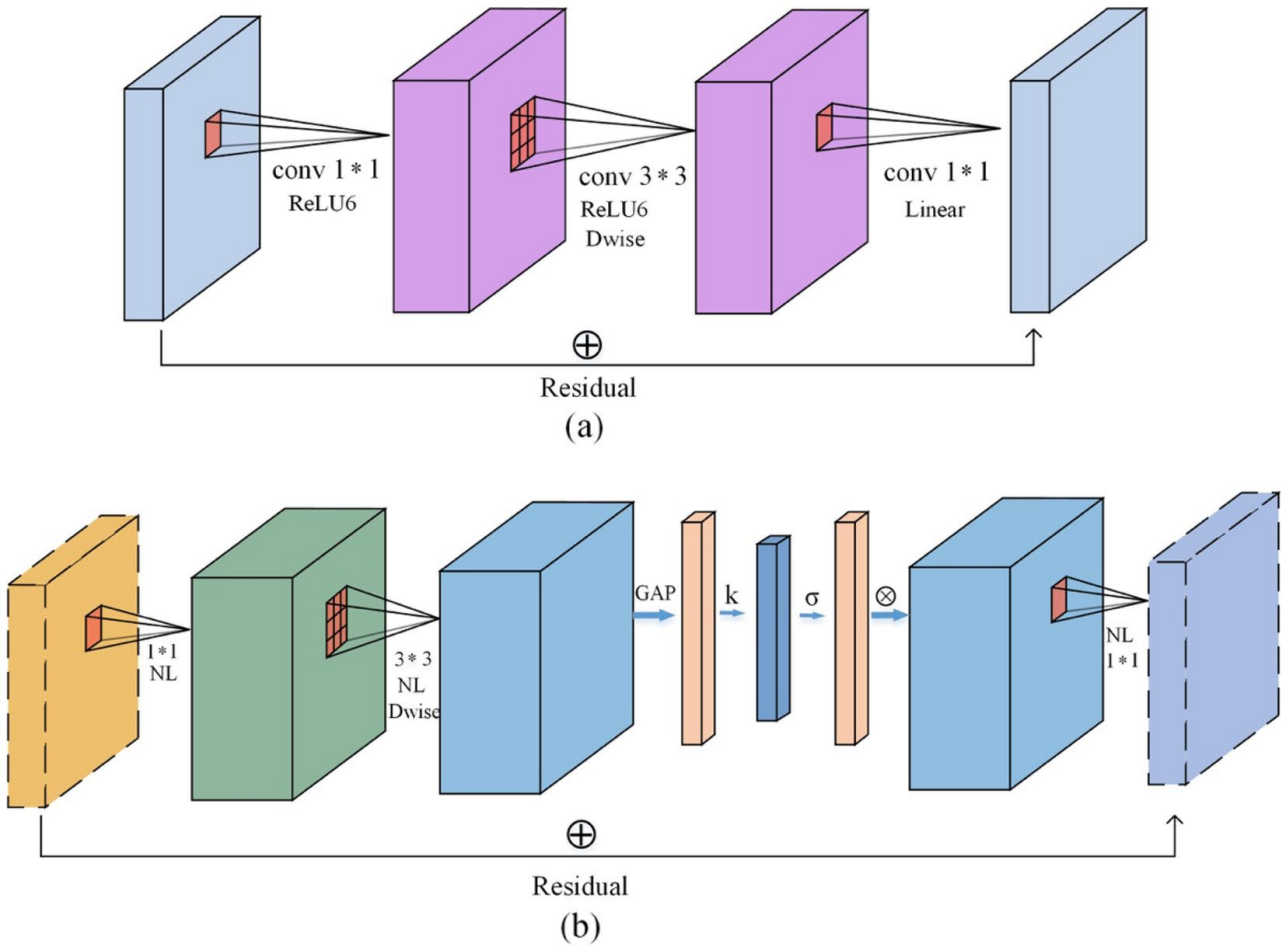
(**a**) Inverted residuals block; (**b**) TF-Bottleneck Block.

In this paper, a TeaFaceNet bottleneck (TF-Bottleneck) block was proposed. This module improved the MobileNetV3 block. Figure 7a shows the inverted residuals block. This block mainly uses ReLU as the activation function. Figure 7b shows the TF-Bottleneck block. The attention block of the ECA module is placed after the depth filter in the extension to facilitate the application of attention to the maximum representation.

#### Backbone feature extraction network

**Figure 8:**
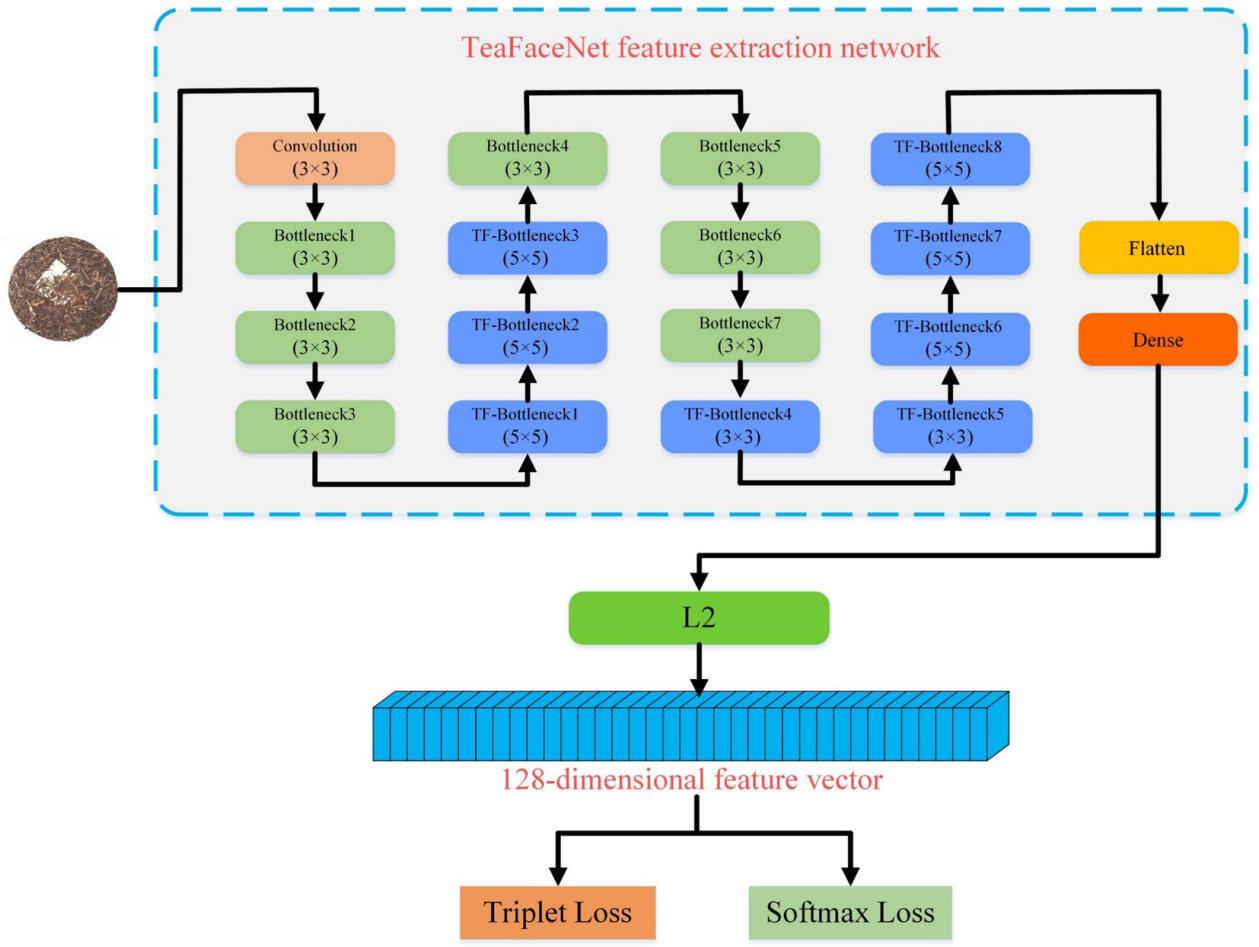
The training structure of the TeaFaceNet model.

TeaFaceNet feeds each batch of data into a redesigned deep convolutional neural network and then performs $L2$ normalization to produce embeddings of tea faces. Both triplet loss and softmax loss are used in training the data, which is eventually used for the tea face verification task. The training structure of the TeaFaceNet model is shown in Fig. 8.

The specifications of the backbone feature extraction network in this paper are shown in Table 3. The initial input size is adjusted to 320 × 320 × 3, and the final output is a 1 × 1 × 128 feature vector. The entire backbone network consists of 18 modules {Layer1, Layer2, Layer3, Layer4, Layer5, …, Layer18}. The {Layer1} includes convolutional, regularization and activation layers, with the activation function using h-swish. {Layer2, Layer3, Layer4} are linear Bottleneck layers, in which no ECA module is added and ReLU is used as the activation function. {Layer5, Layer6, Layer7} are linear TF-Bottleneck layers, and the ECA module is added to these three layers, also using ReLU as the activation function. {Layer8, Layer9, Layer10, Layer11} are linear Bottleneck layers. No ECA module is added to these four layers, and h-swish is used as the activation function. {Layer12, Layer13, Layer14, Layer15, Layer16} are linear TF-Bottleneck layers, in which the ECA module is added and h-swish is used as the activation function. {Layer17} is the Flatten layer, the main purpose of this layer is to flatten the features, which is the transition from the convolutional layer to the fully-connected layer. {Layer18} is a fully-connected neural network layer, whose main purpose is to fully connect the input into a 128-dimensional feature vector.

**Table 3:** Structure of the backbone feature extraction network.

Input	Layer	Filter Size	exp size	out	ECA	NL	stride
320^2^ × 3	Convolution	3 × 3	–	16	False	h-swish	1
320^2^ × 16	Bottleneck1	3 × 3	16	16	False	ReLU	1
320^2^ × 16	Bottleneck2	3 × 3	64	24	False	ReLU	2
160^2^ × 24	Bottleneck3	3 × 3	72	24	False	ReLU	1
160^2^ × 24	TF-Bottleneck1	5 × 5	72	40	True	ReLU	2
80^2^ × 40	TF-Bottleneck2	5 × 5	120	40	True	ReLU	1
80^2^ × 40	TF-Bottleneck3	5 × 5	120	40	True	ReLU	1
80^2^ × 40	Bottleneck4	3 × 3	240	80	False	h-swish	2
40^2^ × 80	Bottleneck5	3 × 3	200	80	False	h-swish	1
40^2^ × 80	Bottleneck6	3 × 3	184	80	False	h-swish	1
40^2^ × 80	Bottleneck7	3 × 3	184	80	False	h-swish	1
40^2^ × 80	TF-Bottleneck4	3 × 3	480	112	True	h-swish	1
40^2^ × 112	TF-Bottleneck5	3 × 3	672	112	True	h-swish	1
40^2^ × 112	TF-Bottleneck6	5 × 5	672	160	True	h-swish	2
20^2^ × 160	TF-Bottleneck7	5 × 5	960	160	True	h-swish	1
20^2^ × 160	TF-Bottleneck8	5 × 5	960	160	True	h-swish	1
20^2^ × 160	Flatten	–	–	–	False	–	–
1^2^ × 64,000	Dense	–	–	128	False	–	–

#### Loss function

**Figure 9:**
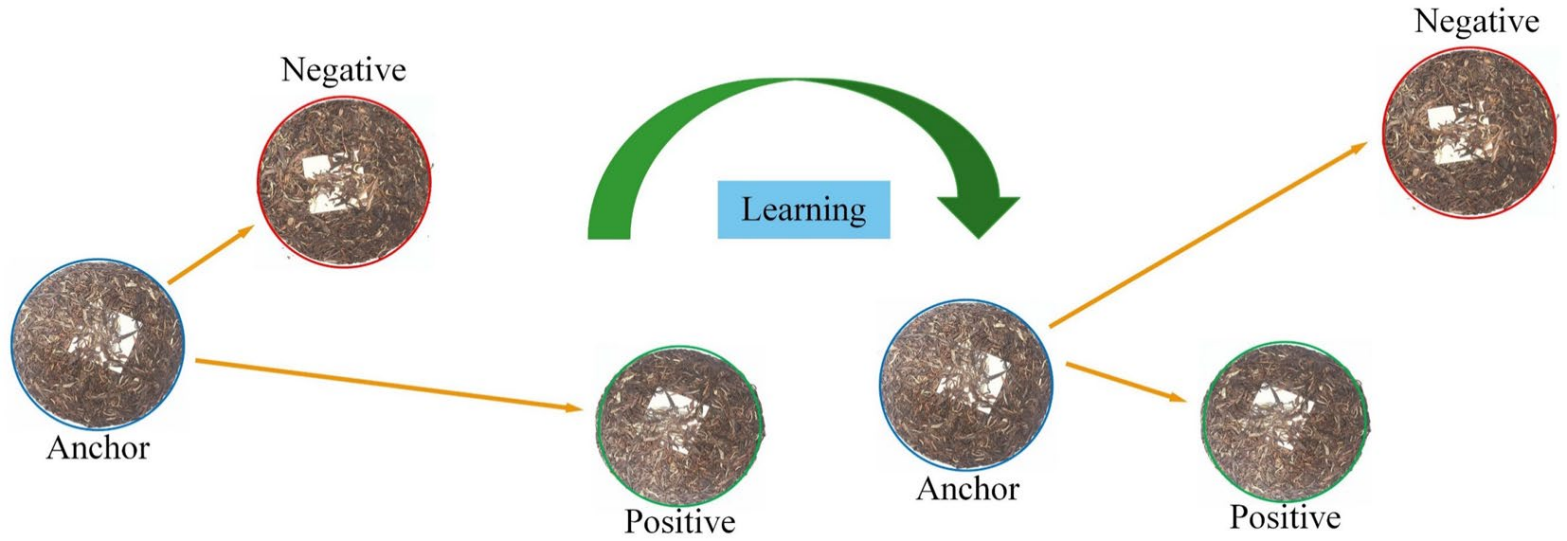
Triplet Loss.

Triplet Loss^28^ is chosen as the main loss function. The main objective is to minimize the Euclidean distance between an anchor and a positive image and maximize the Euclidean distance from a negative image, as shown in Fig. 9. The minimized triplet loss function is shown in Eq. (4),

3 $$  ||x_{i}^{a}  - x_{{i}}^{{p}}||_{2}^{2}  + a < ||x_{i}^{a}  - x_{{i}}^{{n}}||_{2}^{2} ,\forall \left( {x_{i}^{a} ,x_{i}^{p} ,x_{i}^{n} } \right) \in T  $$

4 $$ L_{triplet} = \mathop \sum \limits_{i}^{N} \left[ ||{f\left( {x_{i}^{a} } \right) - f\left( {x_{i}^{p} } \right)||_{2}^{2} - ||f(x_{i}^{a} ) - f(x_{i}^{n} )||_{2}^{2} + a} \right]_{ + } $$

where $a$ increases the distance gap between positive and negative pairs. $T$ is the set of all possible triples in the training set with base $N$.

Meanwhile, softmax loss^11^ is added to the training. Because using only Triplet Loss, the convergence of the model is too slow, which is due to the fact that using triples to select data generates a large number of data sets and the random sampling method is used for selection, which leads to a reduced model training speed. The softmax loss function is shown in Eq. (5),

5 $$ L_{softmax} = - \mathop \sum \limits_{i = 1}^{m} log\frac{{e^{{W_{{y_{i} }}^{T} x_{i} + b_{{y_{i} }} }} }}{{\mathop \sum \nolimits_{j = 1}^{n} e^{{W_{j}^{T} x_{i} + b_{j} }} }} $$

Among them, $x_{i} \in R^{d}$ denotes the $i$ th deep feature, belonging to the $y_{i}$ th class. $d$ is the feature dimension. $W_{j} \in R^{d}$ denotes the $j$ th column of the weights $W_{j} \in R^{d \times n}$ in the last fully connected layer and $b \in R^{n}$ is the bias term. The size of the mini-batch and the number of class is $m$ and $n$.

#### Tea face verification process

**Figure 10:**
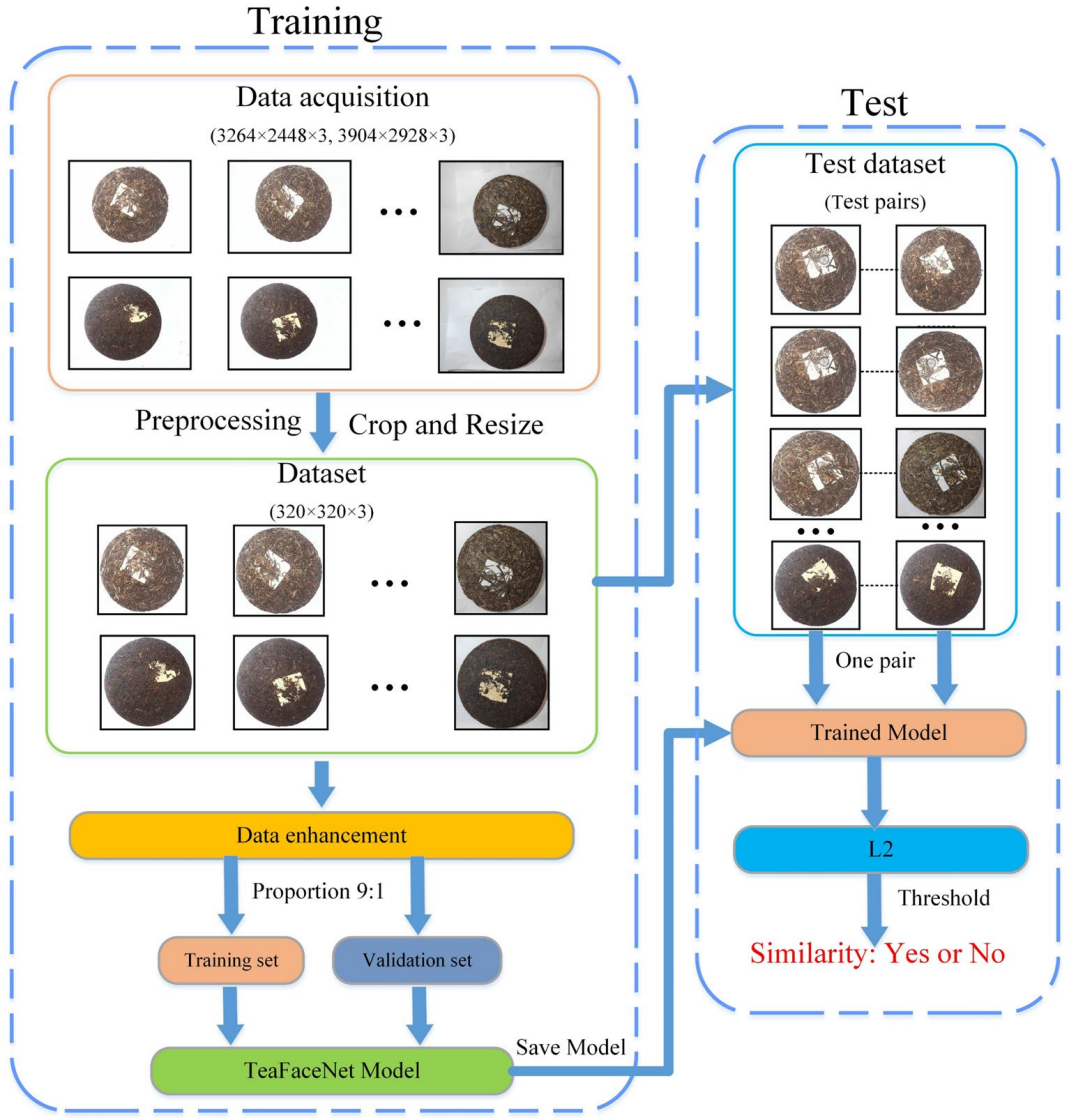
Tea Face Verification Process.

Tea face verification mainly involves inputting two images to be recognized into the trained TeaFaceNet network to extract the depth features of the images and finally form two feature vectors, which are then mapped to a compact Euclidean space. The $L2$ distance between them directly represents the similarity gap between the two tea faces, and the verification result is derived based on the similarity gap threshold, i.e. whether it is the same tea face or not. The specific process of tea face verification is described below, the process is shown in Fig. 10.

1. Crop the dataset image while resizing the image to 320 × 320 × 3.
2. Expand the dataset using image enhancement techniques, including rotation, noise, brightness, chroma, contrast, sharpness adjustment, and random erasing.
3. Divide all the training data into training and validation sets in the ratio of 9:1. Make a test pair of tea face data using the new tea face data.
4. Train the TeaFaceNet model using the training dataset, record the validation set Loss values, and save the model after 100 epochs of training.
5. The images of the test pair are tested by the trained TeaFaceNet model to calculate the L2 distance and get the best threshold.
6. The verification results of the test pair are obtained to achieve tea face verification.

#### Evaluation metrics

To evaluate the performance of the network in the tea face verification datasets, Precision, Recall, F1-Score, and Accuracy are used for performance evaluation. Where $TP$ represents the same data pairs correctly recognized; $TN$ represents different data pairs correctly recognized; $FN$ represents different data pairs incorrectly recognized; $FP$ represents the same data pairs incorrectly recognized. The calculation methods are given in Eq. (6) to Eq. (9),

6 $$ Precision = \frac{TP}{{TP + FP}} \times 100\% $$

7 $$ Recall = \frac{TP}{{TP + FN}} \times 100\% $$

8 $$ F1{-}Score = 2 \times \frac{Precision \times Recall}{{Precision + Recall}} \times 100\% $$

9 $$ Accuracy = \frac{TP + TN}{{TP + FP + TN + FN}} \times 100\% $$

## Results and discussion

### Experimental environment and parameter settings

The experiments were conducted in Python. The code was mainly based on the Keras deep learning framework. TensorFlow was used as the backend. The hardware and software configuration pieces of information are shown in Table 4. The hyperparameters for model training are shown in Table 5.

**Table 4:** Hardware and software configuration information.

Name	Parameter
System	Win10
CPU	Intel(R) Xeon(R) Gold 6130 CPU @ 2.20 GHz
GPU	NVIDIA Quadro P6000
RAM	96 GB
CUDA	10.0.130
TensorFlow	1.13.2
Keras	2.1.5

**Table 5:** Hyperparameters for model training.

Hyperparameters	Value
Epoch	100
Batch Size	30
Optimizer	Adam
Learning Rate	0.001
Decay Rate	0.94
Input Size	320 × 320 × 3

### Tea face recognition results

A test dataset was used to evaluate the TeaFaceNet model. Table 6 shows tea face verification results. The TeaFaceNet was compared with several other mainstream network models, including ResNet50^29^, VGG16^30^, Inception-ResNet-v1^31^, MobileNet and MobileNetV3. Among them, MobileNetV3 had the best recognition effect among the mainstream network models. The recognition accuracy of the raw tea face dataset, ripe tea face dataset and mixed tea face dataset of the TeaFaceNet network were 97.58%, 98.08% and 98.20%, respectively. TeaFaceNet network adds the ECA attention mechanism module to the use of depthwise separable convolution and linear bottlenecks, and the accuracy achieves better results in all three datasets, improving by 1.92%, 2.42% and 0.54% in the three datasets, respectively. The recognition accuracy was improved by replacing the attention mechanism module and redesigning the network structure. In terms of size in the model, TeaFaceNet was only second to MobileNet. The recognition accuracy was improved by 4%, 3% and 1% in the three datasets.

**Table 6:** Tea face verification results.

Model	Accuracy/%			Model Size /MB
	Pu-erh raw tea face	Pu-erh ripe tea face	Mixed tea face	
ResNet50	95.33	93.41	95.91	91.4
VGG16	83.25	92.91	92.50	922
Inception-ResNet-v1	95.58	91.00	94.45	88
MobileNet	93.58	95.08	97.20	**13.1**
MobileNetV3	95.66	95.66	97.66	111
**TeaFaceNet**	**97.58**	**98.08**	**98.20**	**36.9**

Significant are in value [bold].

**Figure 11:**
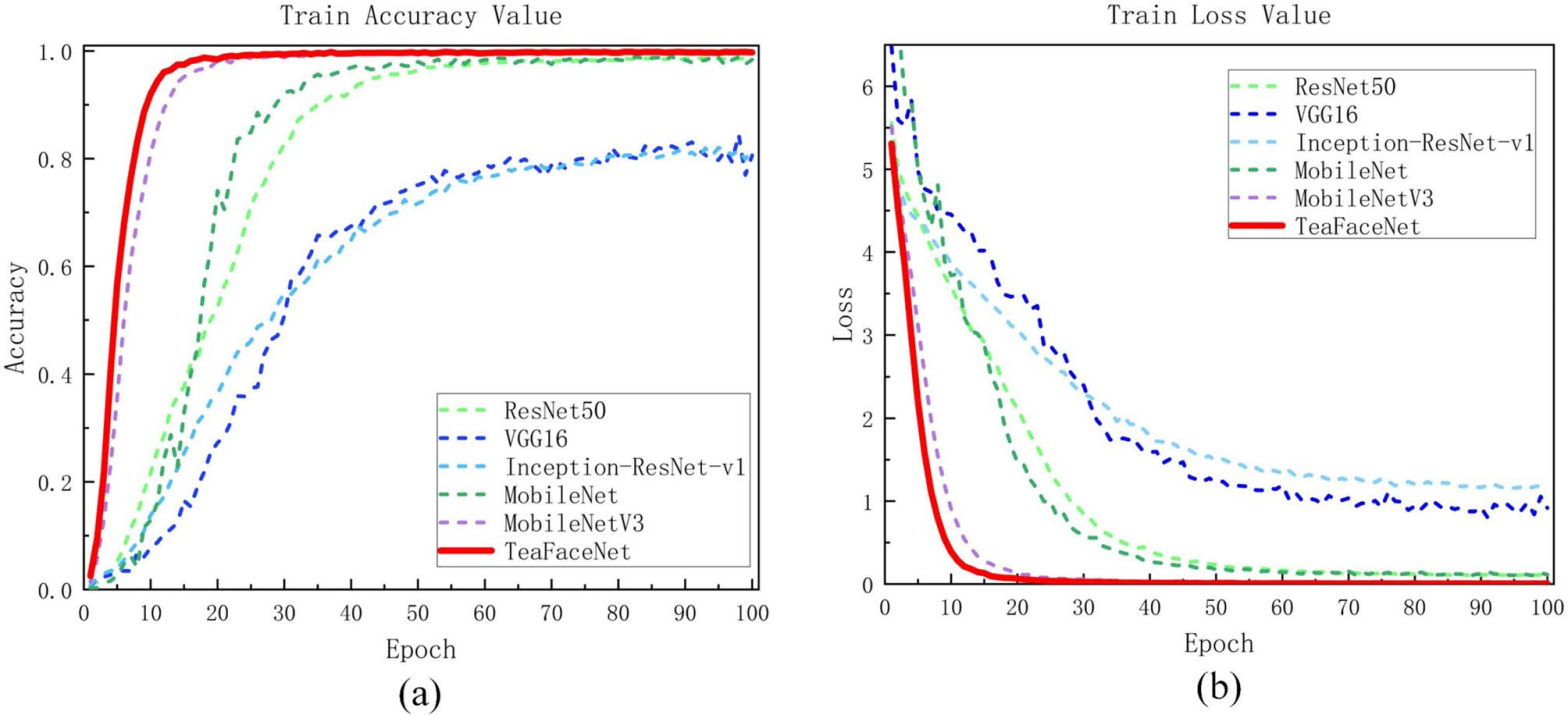
Loss and accuracy of raw tea dataset.

**Figure 12:**
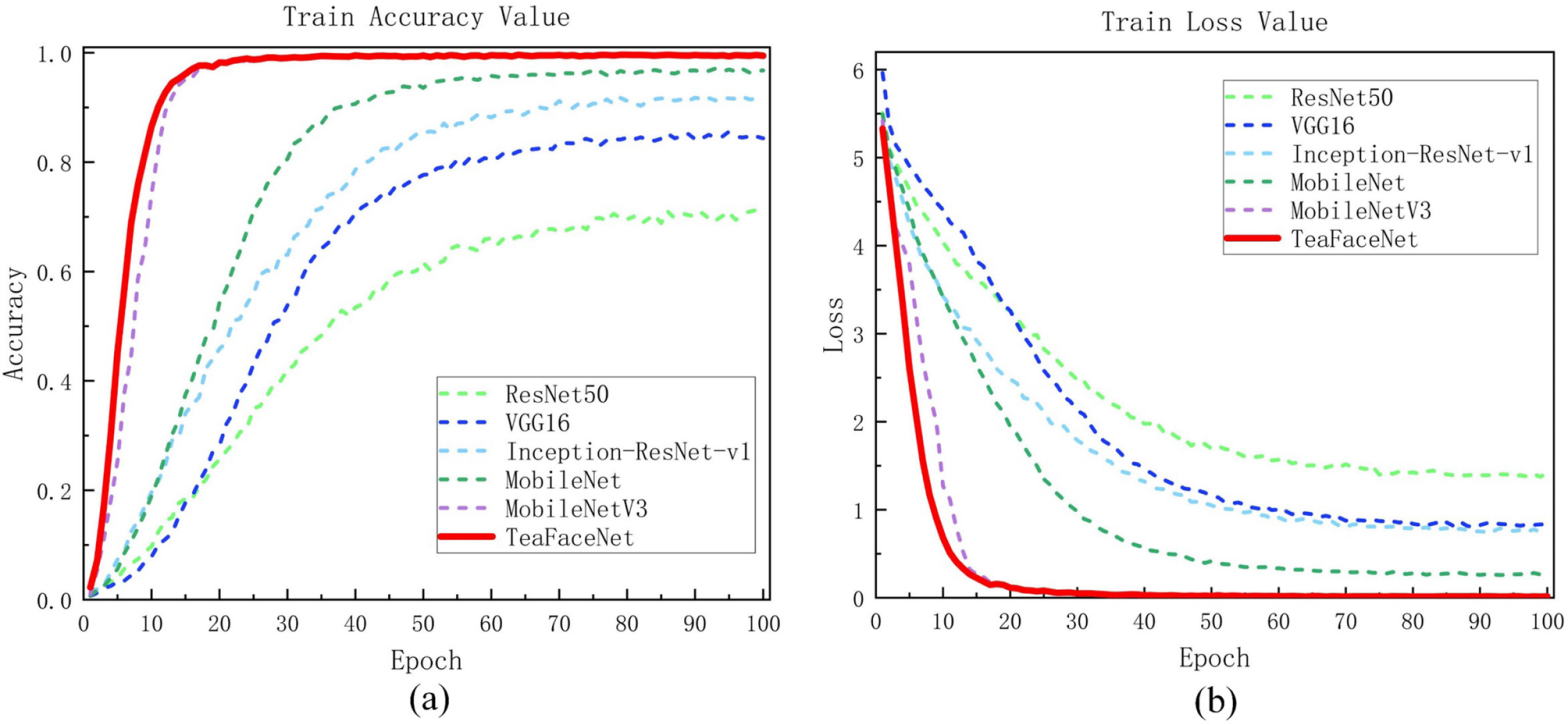
Loss and accuracy of ripe tea dataset.

**Figure 13:**
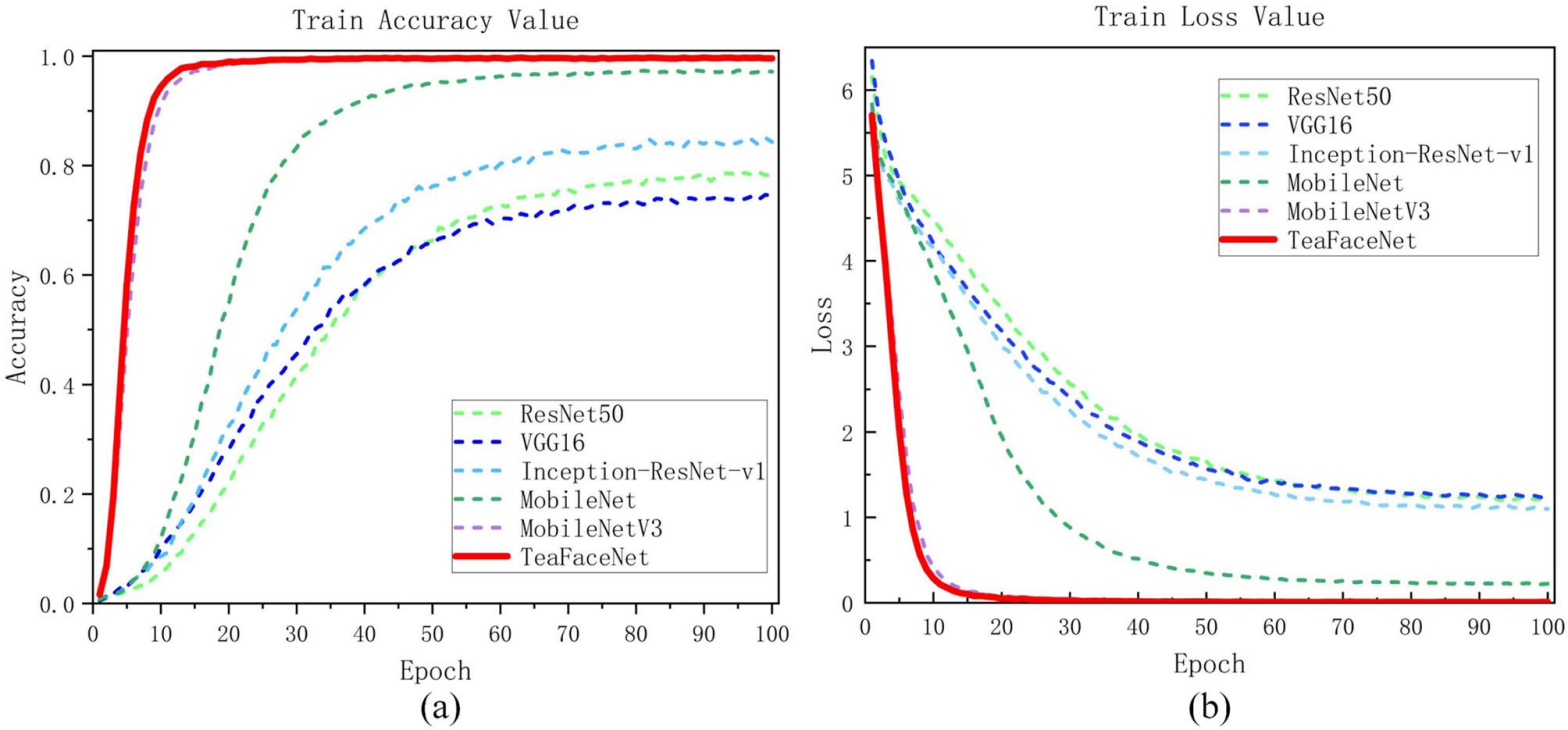
Loss and accuracy of mixed dataset.

TeaFaceNet not only had the best accuracy in the raw tea dataset, mature tea dataset and mixed dataset but also converged first during the model training. A better results could be achieved when the model is trained to 100 epochs. The variation of loss values and validation set accuracy of different network models on the raw tea dataset, ripe tea dataset and mixed dataset are shown in Fig. 11, Fig. 12 and Fig. 13, respectively.

**Figure 14:**
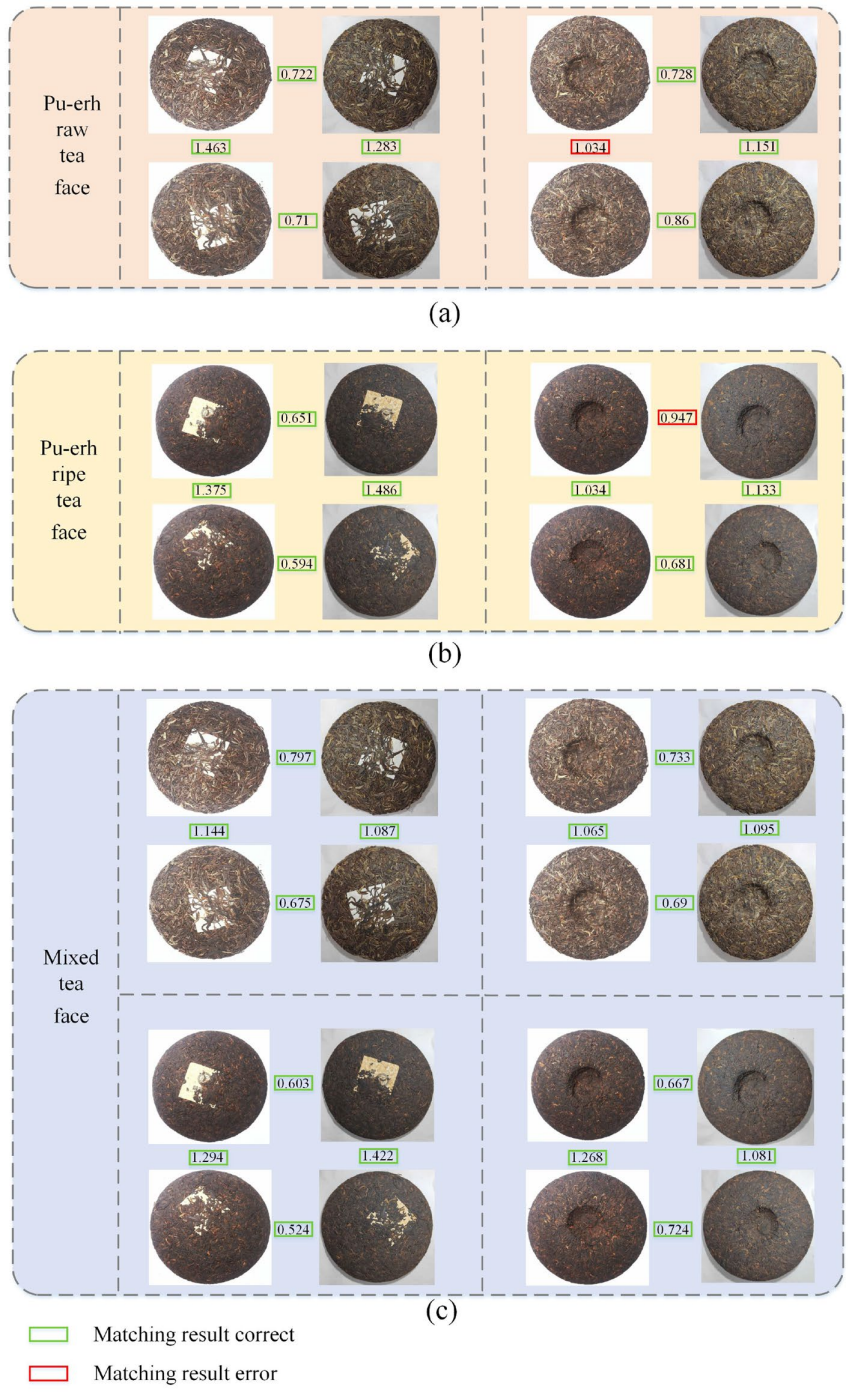
Validation case of TeaFaceNet model.

All tests deal with two main types of problems, i.e., distinguishing between similar tea faces and dissimilar tea faces. Therefore, each model needs to be tested with an optimal threshold. The experiments focus on determining the optimal threshold for each model used ten-fold cross-validation. Table 7 shows the optimal thresholds for all models. The role of the threshold was to determine whether the two tea faces are the similarity. When greater than the optimal threshold, it means that the two tea faces are dissimilar, when less than the optimal threshold, it means that the two tea faces are similar. Figure 14 shows the validation case of the TeaFaceNet model. Where (a) and (b) are the validation results obtained for the model trained using only the raw tea face dataset. (c) and (d) are the validation results obtained for the model trained using only the ripe tea face dataset. (e), (f), (g) and (h) are the validation results obtained for the model trained using the mixed dataset.

**Table 7:** Model optimal thresholds.

Model	Best threshold		
	Pu-erh raw tea face	Pu-erh ripe tea face	Mixed tea face
ResNet50	0.9100	0.7600	0.7000
VGG16	0.9200	0.9700	0.8900
Inception-ResNet-v1	0.8100	0.7000	0.7000
MobileNet	1.0900	0.9400	0.7600
MobileNetV3	1.0400	0.9100	0.9100
**TeaFaceNet**	**1.0600**	**0.9100**	**0.9000**

Significant are in value [bold].

### Model performance analysis

TeaFaceNet improved feature extraction performance and reduced computational effort by introducing the ECA module and using depthwise separable convolution and linear bottlenecks. Compared with the traditional CNNs network, the network achieved a better results in all performances. The Precision, Recall and F1-Score in the raw tea dataset were 97.34%, 97.83% and 97.58%. Compared with MobileNetV3, which increased by 3.29%, 0.33% and 1.84%, respectively. The Precision, Recall and F1-Score in the ripe tea dataset were 98.98%, 97.16% and 98.06%. Compared with MobileNetV3, which increased by 1.91%, 3.00% and 2.47%, respectively. The Precision, Recall and F1-Score in the mixed dataset were 98.82%, 97.58% and 98.20%. Compared to MobileNetV3, which increased by 1.00%, 0.08% and 0.54%, respectively. Table 8 shows the Precision, Recall and F1-Score of the model on the test sets of the raw tea face dataset, ripe tea face dataset and mixed dataset. The experiments showed that TeaFaceNet could be implemented and achieved excellent results on the Pu-erh tea face verification task.

**Table 8:** Model performance analysis.

Model	Datasets	Precision/%	Recall/%	F1-Sorce/%
ResNet50	Pu-erh raw tea face	94.59	96.16	95.37
	Pu-erh ripe tea face	91.54	95.66	93.55
	Mixed tea face	95.46	96.41	95.93
VGG16	Pu-erh raw tea face	78.37	91.83	84.57
	Pu-erh ripe tea face	92.84	93.00	92.92
	Mixed tea face	92.57	92.49	92.49
Inception-Resnet-v1	Pu-erh raw tea face	95.65	95.50	95.57
	Pu-erh ripe tea face	93.92	87.66	90.68
	Mixed tea face	92.64	96.58	94.57
MobileNet	Pu-erh raw tea face	94.39	92.66	93.52
	Pu-erh ripe tea face	96.39	93.66	95.01
	Mixed tea face	96.16	98.33	97.23
MobileNetV3	Pu-erh raw tea face	94.05	97.50	95.74
	Pu-erh ripe tea face	97.07	94.16	95.59
	Mixed tea fac	97.82	97.50	97.66
**TeaFaceNet**	Pu-erh raw tea face	**97.34**	**97.83**	**97.58**
	Pu-erh ripe tea face	**98.98**	**97.16**	**98.06**
	Mixed tea face	**98.82**	**97.58**	**98.20**

Significant are in value [bold].

**Figure 15:**
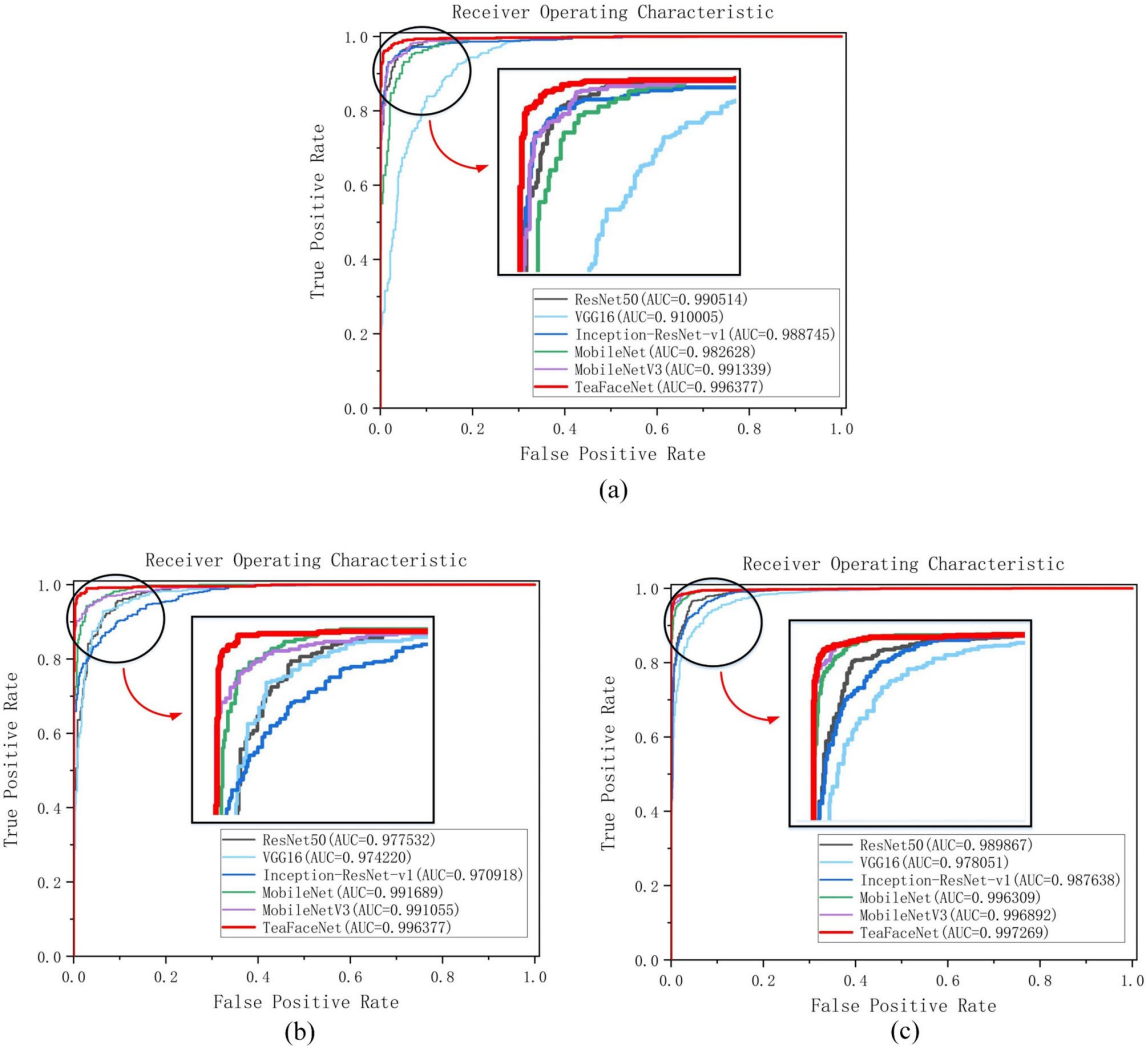
(**a**) ROC curve for raw tea face dataset; (**b**) ROC curve for ripe tea face dataset; (**c**) ROC curve for mixed tea face dataset.

Through the analysis of the receiver operating characteristic (ROC) curve, the quality of the network model could be better determined. The Area Under roc Curve (AUC) value is the size of the part of the area under the ROC curve. The AUC value is between 0.5 and 1.0, with a larger AUC representing better performance. The higher the upper left corner, the better the performance. Figure 15 shows the ROC curves of the model for each of the three datasets. The ROC curves of TeaFaceNet model are in the upper left corner, with AUC values of 0.996377 for raw tea face dataset, 0.996377 for ripe tea face dataset, and 0.997269 for the mixed tea face dataset.

### Effect of attentional mechanism module on the model

To investigate the effect of the attention mechanism module on the model, experiments were conducted by replacing the ECA module in the model with the SE module and the CBAM module. Table 9 shows the results of tea face recognition under different attention mechanism modules. It was shown experimentally that a better results were achieved using the ECA module with the least number of model size. The accuracy increased over the model using the SE module was 0.83%, 0.33%, and 0.25% for the three data sets, and the model size volume was reduced by 5.8 M. The accuracy improvement over the model using the CBAM module was 1.25%, 4.92%, and 2% for the three data sets, and the model size volume was reduced by 72.1 MB. The features between channels in the tea face recognition task had a large impact on the results. It was proven that the ECA module could effectively improve the accuracy of network verification.

**Table 9:** Comparison of tea face recognition results under different attention mechanism modules.

Model	Attention block	Accuracy/%			Model Size/MB
		Pu-erh raw tea face	Pu-erh ripe tea face	Mixed tea face	
0	SE	96.75	97.75	97.95	42.7
1	CBAM	96.33	93.16	96.20	109
**2(ours)**	**ECA**	**97.58**	**98.08**	**98.20**	**36.9**

Significant are in value [bold].

## Discussion

In this work, We propose a Pu-erh tea face verification approach called TeaFaceNet based on an improved MobileNetV3 to enhance Pu-erh tea traceability identification. We construct three types of Pu-erh tea face datasets and establish a Pu-erh tea face verification network to achieve comprehensive verification of Pu-erh raw tea and Pu-erh ripe tea. The TeaFaceNet network achieved recognition accuracies of 97.58%, 98.08%, and 98.20% for the raw tea face dataset, ripe tea face dataset, and mixed tea face dataset, respectively. However, several issues remain in the area of tea face recognition. There is currently no publicly available dataset for Pu-erh tea faces, and the dataset used in this experiment needs further expansion. Our work solely addresses the Pu-erh tea face verification problem, and further exploration is required for the Pu-erh tea face recognition problem. In practical applications, transportation breakage can also pose a challenge, and more discussion is needed for the verification and identification of Pu-erh tea faces after breakage.

## Conclusion

The primary objective of this study was to address the challenge of tracing Pu-erh tea cakes and to facilitate the detection of counterfeit and substandard tea products. In this paper, we proposed a Pu-erh tea face verification model, TeaFaceNet, based on an improved MobileNetV3 architecture. The TeaFaceNet model extracts 128-dimensional features from each pair of Pu-erh tea face images and calculates the L2 distance between them to determine whether they are the same tea face, based on the similarity between images determined by the best threshold. The experimental results demonstrated that the TeaFaceNet model outperformed other models on the Pu-erh tea face dataset. The ECA block reduced the model size while extracting features, thereby improving the recognition rate of the network. The proposed model exhibited better robustness and generalization ability and achieved excellent results not only on individual class tea face verification tasks but also on mixed datasets. Our approach could serve as an empirical basis for subsequent Pu-erh tea face recognition tasks and aid in enhancing the traceability of Pu-erh tea products.

## Abbreviations

AUC: Area under roc curve
CBAM: Convolutional block attention module
CNNs: Convolutional neural networks
ECA: Efficient channel attention
NFC: Near field communication
QR: Quick response
ROC: Receiver operating characteristic
SAM: Spatial attention module
SE: Squeeze-and-excitation
TF-Bottleneck: TeaFaceNet bottleneck
